# Supplementary material for: Haplotype Diversity of NADPH-Cytochrome P450 Reductase Gene of Ophiocordyceps sinensis and the Effect on Fungal Infection in Host Insects
Source: Microorganisms. 2020 Jun 29;8(7):968. doi: 10.3390/microorganisms8070968 (PMC7409138; doi:10.3390/microorganisms8070968)
Supplement: Supplementary file 1 [file microorganisms-08-00968-s001.pdf]

Table S1. Codes, locations, longitudes and latitudes of the 47 *O. sinensis* populations used in this study.

No.	Code	Location	Longitude (E)	Latitude (N)	Altitude (m)	Quantity
1	LSDQ	LasaduilongDeqin,Tibet	91°00′	29°38′	5118	4
2	SNGB	ShannanLuozhaGabu, Tibet	91°46′	29°14′	3820	2
3	SNLZ	ShannanLuozha, Tibet	90°49′	28°11′	3820	5
4	RNML	RekazeNanmulin, Tibet	89°50′	29°40′	4371	5
5	GBZL	GongbujiangdaZhula, Tibet	93°40′	30°00′	3600	5
6	RYD	RekazeYadong, Tibet	88°54′	27°28′	3400	2
7	GBML	GongbujiangdaMila, Tibet	93°15′	29°55′	3820	4
8	LZCY	NyingchiChayu, Tibet	97°28′	28°39′	2300	5
9	LZBJ	NyingchiBujiu, Tibet	94°25′	29°28′	3000	5
10	LZLL	NyingchiLulang, Tibet	94°47′	29°56′	4444	2
11	GBCG	GongbujiangdaCuogao, Tibet	93°53′	29°59′	3600	2
12	GBXK	GongbujiangdaXueka, Tibet	93°41′	29°58′	3600	2
13	NQS	NaquSuoxian, Tibet	93°47′	31°53′	4001	4
14	NQBQ	NaquBaqing, Tibet	94°20′	31°55′	4152	5
15	NQNR	NaquNierong, Tibet	92°17′	32°60′	4777	5
16	NQ	Naqu, Tibet	92°20′	31°28′	4450	5
17	NQBR	NaquBiru, Tibet	93°41′	31°32′	4000	4
18	NQJL	NaquJiali, Tibet	93°13′	30°38′	4500	4
19	LZL	NyingchiLangxian, Tibet	93°40′	29°30′	3520	5
20	LZ	Nyingchi, Tibet	94°15′	29°35′	3000	6
21	ML	NyingchiMilin, Tibet	94°08′	29°11′	3700	8
22	DQ	Dingqing, Tibet	95°38′	31°25′	4300	8
23	DZ	Dazi, Tibet	91°23′	29°37′	3800	2
24	GK	Mozhugongqia, Tibet	91°46′	29°46′	4200	5
25	LL	ChangduLuolong, Tibet	95°45′	30°49′	3640	3
26	YS	Yushu, Qinghai	96°56′	33°02′	4500	6
27	ZD	Zaduo, Qinghai	95°02′	32°55′	4300	7
28	QL	Qilian, Qinghai	100°13′	38°01′	2700	6
29	HZ	Huangzhong, Qinghai	101°34′	36°29′	2260	3
30	GC	Gangcha, Qinghai	100°10′	37°19′	3200	7
31	TJ	Tianjun, Qinghai	99°02′	37°17′	3200	8
32	GH	Gonghe, Qinghai	100°37′	36°16′	3200	7
33	XH	Xinghai, Qinghai	99°59′	35°04′	4300	8
34	GN	Guinan, Qinghai	100°45′	35°34′	3100	7
35	HN	Henan, Qinghai	101°37′	34°45′	3600	7
36	SD	Zhiduo, Qinghai	89°25′	33°21′	4500	2
37	QML	Qumalai, Qinghai	95°30′	34°31′	4176	1
38	DR	Dari, Qinghai	99°41′	33°44′	3968	5
39	MQ	Maqin, Qinghai	100°16′	34°29′	4200	5
40	GD	Gande, Qinghai	99°53′	33°57′	4051	5
41	GUD	Guide, Qinghai	101°28′	36°01′	2238	5
42	TD	Tongde, Qinghai	100°38′	35°14′	3290	3
43	HY	Huangyuan, Qinghai	101°17′	36°43′	2635	5
44	SG	Shangri-La, Yunnan	98°43′	27°47′	4500	3
45	SQ	Shiqu, Sichuan	98°04′	33°01′	4200	6
46	KD	Kangding, Sichuan	101°57′	30°02′	4200	4
47	LQ	Luqu, Gansu	102°30′	34°36′	3106	2
Total						219

Table S2. Primer sequences for amplifying NADPH cytochrome P450 reductase genes of *O. sinensis*.

Gene	Primer	Sequence
NADPH cytochrome P450 reductase-1 1	NADPH-P1F	5'-ACGGAACGGAAAAGAACACG-3'
	NADPH-P1R	5'-CGGGAGTCGCGAGTCAGGTT-3'
NADPH cytochrome P450 reductase-1 2	NADPH-P2F	5'-GGCAACAACACCTACGAGCA-3'
	NADPH-P2R	5'-CCGTCCAGCTCGTAGCTTCC-3'
NADPH cytochrome P450 reductase-1 3	NADPH-P3F	5'-GCAGAAGACGAGCCCTCACT-3'
	NADPH-P3R	5'-ATCTCAGCGCATGCTTCGTT-3'
NADPH cytochrome P450 reductase-2 1	P450-P1F	5'-TCTTGACCTCCTGCATTCCT-3'
	P450-P1R	5'-GCGGTAGAGAAAGTTGGGAG-3'
NADPH cytochrome P450 reductase-2 2	P450-P2F	5'-TCTGTCCATTCGCGGCATGTTC-3'
	P450-P2R	5'-TCCTTGGGCTTGATGGTCAG-3'
NADPH cytochrome P450 reductase-2 3	P450-P3F	5'-AAAGCACACCTTGATCCCGT-3'
	P450-P3R	5'-CGCATCCCACGACAAATTGA-3'
NADPH cytochrome P450 reductase-2 4	P450-P4F	5'-CGAAGATAGCAGTGACAGCG-3'
	P450-P4R	5'-CTGGACGTATTTGCAGCACT-3'
NADPH cytochrome P450 reductase-2 5	P450-P5F	5'-ACCAGCTTTCTCTCCGGTCT-3'
	P450-P5R	5'-GCTCACAACTCGACACTCGT-3'

Table S3. PCR reaction system of NADPH cytochrome P450 reductase genes of *O. sinensis*.

**Reagent**	**Volume（μL）**
10× Buffer（Mg^{2+} free）	5
dNTPs（10 mmol/L）	1.25
$MgCl_2$（25 mmol/L）	4
Primer F（10 mmol/L）	2.5
Primer R（10 mmol/L）	2.5
Ex TaqDNA polymerase（5 U/μL）	0.5
DNA（20 ng/μL）	2.5
ddH_2O	31.75
Total	50

Table S4. GenBank accession numbers of NADPH cytochrome P450 reductase-1 gene sequence obtained in this study.

**Haplotype**	**GenBank accession No.**	**Haplotype**	**GenBank accession No.**
H1	MT130777	H15	MT130791
H2	MT130778	H16	MT130792
H3	MT130779	H17	MT130793
H4	MT130780	H18	MT130794
H5	MT130781	H19	MT130795
H6	MT130782	H20	MT130796
H7	MT130783	H21	MT130797
H8	MT130784	H22	MT130798
H9	MT130785	H23	MT130799
H10	MT130786	H24	MT130780
H11	MT130787	H25	MT130781
H12	MT130788	H26	MT130782
H13	MT130789	H27	MT130783
H14	MT130790	H28	MT130804

Table S5. GenBank accession numbers of NADPH cytochrome P450 reductase-2 gene sequence obtained in this study.

**Haplotype**	**GenBank accession No.**	**Haplotype**	**GenBank accession No.**
H1	MT130805	H16	MT130820
H2	MT130806	H17	MT130821
H3	MT130807	H18	MT130822
H4	MT130808	H19	MT130823
H5	MT130809	H20	MT130824
H6	MT130810	H21	MT130825
H7	MT130811	H22	MT130826
H8	MT130812	H23	MT130827
H9	MT130813	H24	MT130828
H10	MT130814	H25	MT130829
H11	MT130815	H26	MT130830
H12	MT130816	H27	MT130831
H13	MT130817	H28	MT130832
H14	MT130818	H29	MT130833
H15	MT130819		

Table S6. GenBank accession numbers of COI gene sequences of host insects of *O. sinensis* obtained in this study.

Haplotype	GenBank accession No.	Haplotype	GenBank accession No.
H1	KC994913	H34	KC994946
H2	KC994914	H35	KC994947
H3	KC994915	H36	KC994948
H5	KC994917	H39	KC994951
H6	KC994918	H40	KC994952
H7	KC994919	H42	KC994954
H8	KC994920	H43	KC994955
H9	KC994921	H44	KC994956
H10	KC994922	H46	KC994958
H11	KC994923	H47	KC994959
H12	KC994924	H53	KC994965
H13	KC994925	H56	KC994968
H14	KC994926	H62	KC994974
H15	KC994927	H63	KC994975
H16	KC994928	H65	KC994977
H17	KC994929	H67	KC994979
H19	KC994931	H68	KC994980
H20	KC994932	H69	KC994981
H22	KC994934	H70	KC994982
H23	KC994935	H71	KC994983
H24	KC994936	H72	KC994984
H25	KC994937	H73	KC994985
H26	KC994938	H74	KC994986
H27	KC994939	H75	KC994987
H28	KC994940	H76	KC994988
H29	KC994941	H80	KC994992
H30	KC994942	H81	KC994993
H31	KC994943	H82	KC994994
H32	KC994944	H85	KC994997
H33	KC994945		

Table S7. Haplotype correspondence of host insect COI gene, NADPH CPR-1 gene,. and NADPH CPR-2 gene of *O. sinensis* of all individuals

Population No.	Population code	Individual code	COI	NADPH CPR-1	NADPH CPR-2
1	LSDQ	LSDQ-1	H1	H2	H2
		LSDQ-2	H1	H3	H2
		LSDQ-3	H1	H1	H3
		LSDQ-4	H1	H1	H2
2	SNGB	SNGB-2	H3	H4	H1
		SNGB-4	H2	H3	H1
3	SNLZ	SNLZ-1	H5	H5	H4
		SNLZ-2	H6	H6	H5
		SNLZ-3	H5	H7	H5
		SNLZ-4	H6	H3	H4
		SNLZ-5	H6	H8	H4
4	RNML	RNML-1	H1	H1	H2
		RNML-2	H1	H3	H2
		RNML-3	H1	H3	H3
		RNML-4	H1	H1	H1
		RNML-5	H7	H1	H1
5	GBZL	GBZL-1	H8	H1	H1
		GBZL-2	H9	H9	H6
		GBZL-3	H10	H10	H7
		GBZL-4	H9	H11	H7
		GBZL-5	H9	H10	H8
6	RYD	RYD-4	H11	H12	H5
		RYD-5	H11	H12	H5
7	GBML	GBML-1	H12	H1	H1
		GBML-2	H12	H13	H9
		GBML-3	H13	H11	H6
		GBML-4	H14	H1	H1
8	LZCY	LZCY-1	H15	H1	H1
		LZCY-2	H15	H1	H1
		LZCY-3	H15	H1	H1
		LZCY-4	H16	H13	H7
		LZCY-5	H15	H1	H1
9	LZBJ	LZBJ-1	H17	H14	H10
		LZBJ-2	H17	H15	H11
		LZBJ-3	H17	H1	H11
		LZBJ-4	H17	H16	H10
		LZBJ-5	H17	H17	H12
10	LZLL	LZLL-4	H19	H18	H11
		LZLL-5	H19	H18	H11
11	GBCG	GBCG-1	H20	H1	H11
		GBCG-5	H22	H1	H13
12	GBXK	GBXK-2	H23	H1	H14
		GBXK-3	H1	H1	H14
13	NQS	NQS-1	H24	H1	H1
		NQS-2	H15	H1	H1
		NQS-3	H15	H1	H1
		NQS-5	H26	H1	H1
14	NQBQ	NQBQ-1	H15	H1	H1
		NQBQ-2	H15	H1	H1
		NQBQ-3	H15	H1	H15
		NQBQ-4	H15	H1	H1
		NQBQ-5	H27	H1	H1
15	NQNR	NQNR-1	H15	H1	H1

		NQNR-2	H15	H1	H1
		NQNR-3	H28	H1	H1
		NQNR-4	H15	H1	H1
		NQNR-5	H15	H1	H1
16	NQ	NQ-1	H29	H1	H1
		NQ-2	H28	H1	H1
		NQ-3	H28	H1	H1
		NQ-4	H15	H1	H1
		NQ-5	H15	H1	H1
17	NQBR	NQBR-1	H15	H1	H1
		NQBR-2	H15	H1	H1
		NQBR-3	H24	H1	H1
		NQBR-4	H30	H1	H1
18	NQJL	NQJL-1	H15	H1	H1
		NQJL-3	H31	H1	H1
		NQJL-4	H15	H1	H1
		NQJL-5	H15	H1	H1
19	LZL	LZL-1	H1	H11	H7
		LZL-2	H1	H13	H7
		LZL-3	H32	H13	H7
		LZL-4	H33	H1	H1
		LZL-5	H34	H13	H16
20	LZ	LZ-1	H62	H22	H1
		LZ-3	H63	H1	H7
		LZ-6	H65	H18	H1
		LZ-7	H66	H1	H22
		LZ-8	H66	H1	H22
		LZ-9	H62	H1	H1
21	ML	ML-3	H69	H1	H1
		ML-4	H70	H23	H11
		ML-5	H67	H23	H1
		ML-6	H71	H1	H24
		ML-7	H68	H23	H1
		ML-8	H72	H23	H22
		ML-9	H72	H23	H22
		ML-10	H67	H17	H25
22	DQ	DQ-1	H15	H1	H1
		DQ-2	H15	H1	H11
		DQ-3	H15	H1	H1
		DQ-4	H24	H1	H7
		DQ-7	H15	H1	H1
		DQ-8	H56	H1	H22
		DQ-9	H56	H1	H17
		DQ-10	H15	H1	H15
23	DZ	DZ-9	H1	H1	H2
		DZ-10	H1	H1	H1
24	GK	GK-2	H73	H26	H11
		GK-4	H74	H1	H1
		GK-5	H75	H27	H1
		GK-6	H76	H28	H29
		GK-7	H74	H27	H11
25	LL	LL-2	H15	H1	H1
		LL-3	H53	H1	H1
		LL-4	H15	H1	H1
26	YS	YS-4	H26	H1	H1
		YS-6	H26	H1	H1
		YS-7	H26	H1	H1

		YS-8	H26	H1	H1
		YS-9	H36	H1	H1
		YS-10	H26	H1	H1
27	ZD	ZD-3	H44	H1	H1
		ZD-4	H15	H1	H1
		ZD-5	H15	H1	H1
		ZD-6	H15	H1	H1
		ZD-7	H15	H1	H1
		ZD-8	H15	H1	H1
		ZD-10	H15	H1	H1
28	QL	QL-2	H81	H1	H17
		QL-5	H81	H1	H17
		QL-6	H81	H1	H17
		QL-8	H81	H1	H17
		QL-9	H81	H1	H17
		QL-10	H81	H1	H17
29	HZ	HZ-3	H35	H1	H18
		HZ-4	H35	H1	H1
		HZ-5	H35	H1	H1
30	GC	GC-3	H81	H1	H17
		GC-4	H81	H1	H19
		GC-5	H81	H1	H19
		GC-6	H81	H1	H20
		GC-7	H81	H1	H19
		GC-8	H81	H1	H19
		GC-9	H81	H1	H17
31	TJ	TJ-3	H81	H1	H17
		TJ-4	H81	H1	H17
		TJ-5	H81	H1	H19
		TJ-6	H81	H1	H19
		TJ-7	H81	H1	H17
		TJ-8	H81	H1	H19
		TJ-9	H81	H19	H21
		TJ-10	H81	H20	H19
32	GH	GH-4	H81	H1	H17
		GH-5	H81	H1	H17
		GH-6	H81	H1	H19
		GH-7	H81	H1	H19
		GH-8	H81	H1	H19
		GH-9	H81	H1	H19
		GH-10	H81	H1	H17
33	XH	XH-3	H35	H1	H1
		XH-4	H26	H1	H17
		XH-5	H26	H1	H17
		XH-6	H43	H1	H1
		XH-7	H26	H1	H1
		XH-8	H46	H1	H11
		XH-9	H80	H1	H1
		XH-10	H35	H1	H7
34	GN	GN-3	H26	H1	H1
		GN-4	H25	H1	H17
		GN-5	H26	H1	H22
		GN-6	H26	H1	H1
		GN-7	H26	H1	H1
		GN-8	H43	H1	H11
		GN-9	H26	H1	H1
35	HN	HN-3	H26	H1	H23

		HN-4	H26	H21	H1
		HN-5	H26	H1	H22
		HN-6	H26	H1	H22
		HN-7	H26	H1	H1
		HN-8	H26	H1	H1
		HN-9	H26	H1	H11
36	SD	SD-3	H26	H1	H17
		SD-4	H42	H1	H22
37	QML	QML-9	H26	H1	H1
38	DR	DR-3	H26	H1	H11
		DR-4	H26	H1	H27
		DR-5	H26	H1	H1
		DR-6	H26	H1	H1
		DR-7	H26	H1	H1
39	MQ	MQ-1	H26	H1	H17
		MQ-2	H26	H1	H1
		MQ-3	H26	H1	H1
		MQ-4	H26	H1	H11
		MQ-5	H26	H1	H1
40	GD	GD-4	H26	H1	H1
		GD-5	H26	H1	H1
		GD-6	H26	H1	H1
		GD-9	H26	H1	H1
		GD-10	H39	H1	H1
41	GUD	GUD-3	H35	H1	H1
		GUD-4	H26	H1	H1
		GUD-5	H35	H1	H28
		GUD-6	H35	H24	H11
		GUD-7	H26	H1	H1
42	TD	TD-4	H35	H1	H18
		TD-5	H35	H1	H1
		TD-6	H40	H1	H19
43	HY	HY-1	H35	H1	H1
		HY-3	H35	H1	H1
		HY-4	H35	H1	H1
		HY-7	H35	H1	H1
		HY-8	H35	H25	H1
44	SG	SG-4	H82	H13	H1
		SG-5	H85	H13	H1
		SG-6	H85	H13	H1
45	SQ	SQ-2	H26	H1	H1
		SQ-4	H26	H1	H11
		SQ-6	H26	H1	H1
		SQ-7	H26	H1	H1
		SQ-8	H26	H1	H1
		SQ-10	H26	H1	H22
46	KD	KD-2	H47	H1	H17
		KD-4	H47	H1	H1
		KD-7	H47	H13	H26
		KD-10	H47	H1	H11
47	LQ	LQ-9	H26	H1	H1
		LQ-10	H80	H1	H1
